# Occupational exposures and small airway obstruction in the UK Biobank Cohort

**DOI:** 10.1183/23120541.00650-2022

**Published:** 2023-05-22

**Authors:** Johanna Feary, Valentina Quintero-Santofimio, James Potts, Roel Vermeulen, Hans Kromhout, Ben Knox-Brown, Andre F.S. Amaral

**Affiliations:** 1 National Heart and Lung Institute, Imperial College London, London, UK; 2 Institute for Risk Assessment Sciences, University of Utrecht, Utrecht, The Netherlands

## Abstract

**Background:**

Small airways obstruction (SAO) is a key feature of both COPD and asthma, which have been associated with workplace exposures. Whether SAO, which may occur early in the development of obstructive lung disease and without symptoms, also associates with occupational exposures is unknown.

**Methods:**

Using UK Biobank data, we derived measurements of SAO from the 65 145 participants with high-quality spirometry and lifetime occupational histories. The ALOHA+ Job Exposure Matrix was used to assign lifetime occupational exposures to each participant. The association between SAO and lifetime occupational exposures was evaluated using a logistic regression model adjusted for potential confounders. A second logistic regression model was also run to account for potential co-exposures.

**Results:**

SAO was present in varying proportions of the population depending on definition used: 5.6% (forced expiratory flow between 25 and 75% of the forced vital capacity (FEF_25–75_) < lower limit of normal (LLN)) and 21.4% (forced expiratory volume in 3 s (FEV_3_)/forced expiratory volume in 6 s (FEV_6_) <LLN). After adjustment for confounders and co-exposures, people in the highest category of exposure to pesticides were significantly more likely to have SAO (FEV_3_/FEV_6_ <LLN: OR 1.24, 95% CI 1.06–1.44). The association between pesticides and SAO showed an exposure-response pattern. SAO was also less likely among people in the highest exposure categories of aromatic solvents (FEV_3_/FEV_6_ <LLN: OR 0.85, 95% CI 0.73–0.99) and metals (FEV_3_/FEV_6_ <LLN: OR 0.77, 95% CI 0.62–0.94).

**Conclusion:**

Our findings suggest that occupational exposure to pesticides play a role in the SAO. However, further work is needed to determine causality, and identify the specific component(s) responsible and the underlying mechanisms involved.

## Abstract 2

**A history of occupational exposure to pesticides, particularly at high levels, may contribute to the development of small airway obstruction. This relationship is independent of age, sex, cigarette smoking and socioeconomic status.** https://bit.ly/3TkmSKQ

## Introduction

An estimated 15% of COPD and of adult-onset asthma may be caused by workplace respiratory exposures [1]. A key feature of COPD and some asthma is damage to the small airways (<2 mm diameter), which show a dramatic increase in resistance [2]. Studies in ever-smokers and hospital-based populations suggest that small airways obstruction (SAO) precedes spirometric evidence of COPD, radiological detection of emphysema and asthma diagnosis [3–6]. Identification of early subclinical SAO provides an opportunity to intervene with assessment of and, if appropriate, interventions to reduce harmful workplace exposures that may be contributing to the development of respiratory diseases.

Small airways function can be assessed using different methods, including body plethysmography, impulse oscillometry or forced oscillation technique, nitrogen wash out, high-resolution computed tomography, hyperpolarised magnetic resonance imaging and spirometry.

Evaluation of lung health through spirometry is a cornerstone of occupational health surveillance and of clinical evaluation in the healthcare setting. With the growing interest in the life course evolution of lung function, there has been a resurgence of interest in the potential of mid-expiratory flow (MEF) rates, measured by spirometry, to identify early subclinical SAO amongst those who would otherwise be considered to be healthy on the basis of having a ratio of forced expiratory volume in 1 s (FEV_1_) and forced vital capacity (FVC) (FEV_1_/FVC) comparable to population reference equations [7–10]. Conversely, Quanjer *et al.* [11] showed that there was little discordance between airway obstruction diagnosed by the use of FEV_1_/FVC compared to the use of MEF rates, with the greatest difference between the two measures observed in younger age groups. Because of the scarce and limited research conducted on this topic, clinicians rarely consider low MEFs in isolation as relevant in the diagnostic process. As a result, recent research has focused on the use of the FEV in 3 s as a ratio of the FVC (FEV_3_/FVC) or of the FEV in 6 s (FEV_3_/FEV_6_) to detect SAO [6, 12]. Both of these ratios account for variation in the FVC and are therefore subject to less measurement error then the MEF.

There is limited evidence on the association between occupational exposures and SAO. Small occupational studies have reported associations of SAO with particular exposures such as, for example, fungi and mineral dust [13]. However, to date, these studies have been restricted to working populations, which can introduce bias from healthy worker effect. Two European population-based studies, one in the Netherlands and one in Spain, have reported associations of SAO with workplace exposures to vapours, gases, dusts and fumes [14, 15]. However, neither investigated which specific occupational exposures put workers at increased risk of SAO.

Here, we use the UK Biobank population cohort, which comprises the largest dataset of lung function measurements collected in the world, to identify occupational risk factors for SAO using a job exposure matrix to assign occupational exposures and to assess longitudinal changes in SAO.

## Methods

### The UK Biobank population cohort

UK Biobank recruited over 500 000 participants aged 40–69 years from 22 different centres across England, Wales and Scotland, between 2006 and 2010 [16]. Following written informed consent, participants completed a baseline assessment consisting of a detailed questionnaire, recording of self-reported medical history, and clinical assessment which included spirometry measurement. Records were obtained for 502 414 individuals. The study was approved by the UK National Research Ethics Service Committee North West – Haydock.

### Definition of SAO

Spirometry was performed using a calibrated Vitalograph Pneumotrac 6800. Quality control was conducted on the spirometry data following previously described criteria [17]. In brief, to have the highest quality spirometry, participants had to have a minimum of two spirograms fulfilling all the following criteria: no cough, back-extrapolated volume <5% FVC (or >5% but <150 mL), reproducible FEV_1_ and FVC, and a forced expiratory time of ≥6 s on the best curve (curve with highest FEV_1_ and FVC).

Spirometry-derived data were readily available for FEV_1_ and FVC in the UK Biobank. We also extracted from the raw data the values for FEV_3_ and FEV_6_ and mean forced expiratory flow between the 25% and 75% of the FVC (FEF_25–75_) which were required to define SAO. Owing to lack of literature agreement on which spirometry parameter is best to define SAO [18], three different spirometry parameters (FEF_25–75_, FEV_3_/FVC and FEV_3_/FEV_6_) were considered, *a priori*, in the analysis. When we explored the data further, we found that the mean FEV_6_ and FVC values were very similar (3.74 L and 3.77 L respectively) suggesting that the FVC is likely to be underestimated (as a result of the spirometry protocol used in UK Biobank). This leads to a falsely elevated FEV_3_/FVC ratio and an apparent underestimation of the prevalence of SAO. We therefore chose to define SAO as FEF_25–75_ or FEV_3_/FEV_6_ below the lower limit of normal (LLN), based on Hankinson *et al.* 1999 [19] and Hansen 2014 *et al.* [20] reference equations.

### Lifetime occupational exposures

Participants in the UK Biobank self-coded their job using a three-level tree categorisation system, OSCAR (Occupations Self-Coding Automatic Recording), based on the Standard Occupational Classification 2000 (SOC2000) [21] to identify each paid job held for >6 months [22]. In the UK Biobank, the job code is reported as a six-digit number, where the first four digits correspond to the SOC2000. To assign lifetime occupational respiratory exposures to each UK Biobank participant, the ALOHA+ Job Exposure Matrix (JEM) was used. The ALOHA+ JEM is based on expert assessment by industrial hygienists. It assigns three levels of exposure (0, none; 1, low; 2, high) based on the International Standard Classification of Occupations V.1988 (ISCO-88) and on the probability of exposure to 10 different agents in each job (biological dusts, mineral dusts, gases and fumes, herbicides, insecticides, fungicides, aromatic solvents, chlorinated solvents, other solvents and metals) and two composite categories (VDGF: vapours, dusts, gases and fumes; and “all pesticides”: herbicides, insecticides, fungicides) [23]. The official matching key from the UK Office for National Statistics [21] was used to cross-map SOC2000 and the ISCO-88 before assigning the JEM to each participant.

Cumulative exposure for each occupational exposure agent was calculated using duration of each job and the squared intensity of exposure (0, 1 or 4). This variable was expressed in exposure unit-years (EU-years) and categorised as never exposed, low (<median), moderate (≥median to <90th percentile) and high (≥90th percentile) exposure. This provided a lifetime exposure history for each occupational agent for every participant.

### Statistical analysis

**FIGURE 1:**
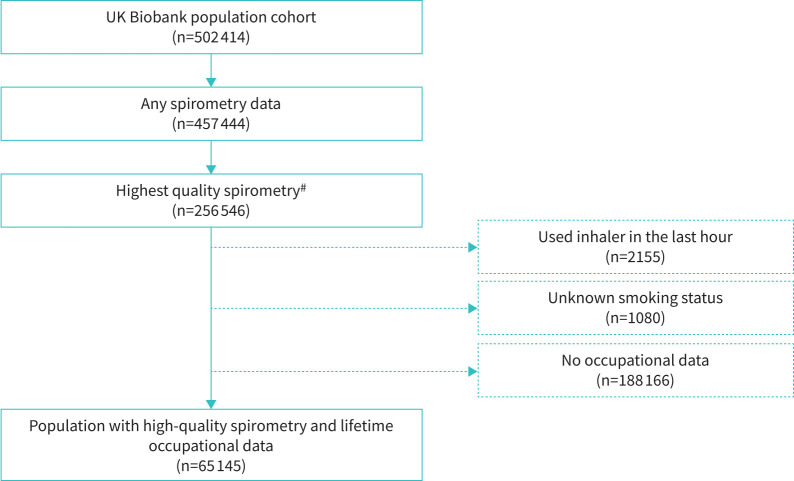
Flow diagram showing selection of final population for analysis. ^#^: criteria for highest quality spirometry: at least two spirograms with no cough, back extrapolated volume <5% FVC (or >5% but <150 mL), reproducible FEV_1_ and FVC and a forced expiratory time ≥6 s on the best curve (curve with highest FEV_1_ and FVC). FEV_1_: forced expiratory volume in 1 s; FVC: forced vital capacity.

We excluded participants with no available spirometry records (n=44 970), those without high-quality spirometry (n=200 898), those who had used an inhaler in the preceding hour (n=2155), those with unknown smoking status (n=1080) and those who did not complete the OSCAR questionnaire on occupation (n=188 166). Baseline questionnaire data, lifetime occupational histories and high-quality spirometry data were available for 65 145 individuals (figure 1).

The association between SAO and lifetime occupational exposures was evaluated using a logistic regression model adjusted for various potential confounders determined *a priori*: age, sex, smoking status (never, ex-smoker or current smoker), smoking pack-years, UK Biobank assessment centre (n=22), Townsend deprivation index (based on small area data) and ethnicity (white *versus* non-white). Results were reported as odds ratios (OR) and 95% confidence intervals (CI). A threshold of significance of 0.05 was considered. A second logistic regression model was run to account for potential co-exposures and minimise collinearity, with adjustments made for significant associations determined in the previous model. Analyses were carried out using RStudio (version 2021.09.0–351) [24] and R (version 4.1.1) [25].

## Results

**FIGURE 2:**
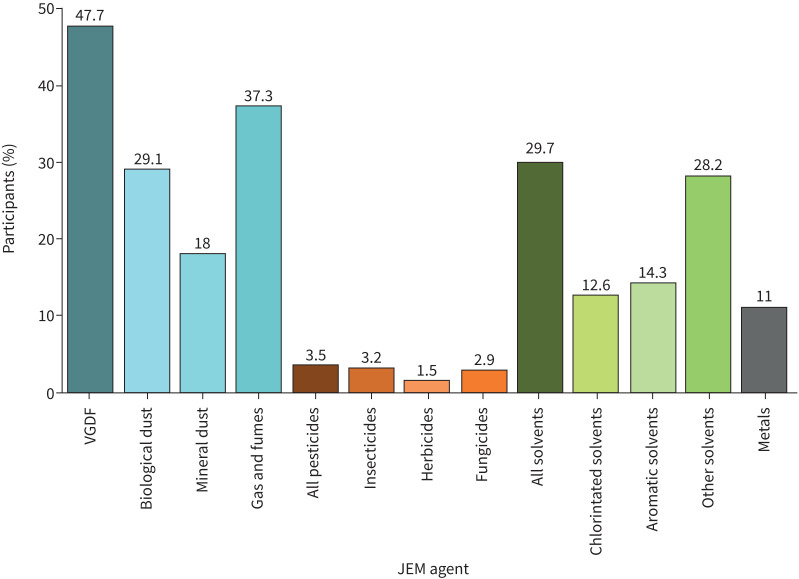
Participants in the included UK Biobank study population (n=65 145) with cumulative exposure to one or more JEM agents. JEM: Job Exposure Matrix; VDGF: vapour, dust, gases and fumes.

The majority of the 65 145 participants included in this study were female (60.3%), 94.4% were of white ancestry, 61% had never smoked and the median Townsend deprivation index was −2.47 reflecting relative affluence. Women were more likely to have never smoked than men (61% *versus* 52%). In total, airflow obstruction (FEV_1_/FVC <LLN) was present in 8%. SAO was present in varying proportions of the population depending on the definition used: 5.6% using FEF_25–75_ <LLN and 21.4% using FEV_3_/FEV_6_ <LLN (table 1). When the population was restricted to those who had never smoked and who had no history of exposure to the occupational agents of interest, the prevalence estimates of SAO were slightly lower: 4.7% using FEF_25–75_ <LLN and 19.2% using FEV_3_/FEV_6_ <LLN. Half the population had been exposed to at least one occupational agent of interest (48.5%); 16.5% were exposed to a single agent, 21.4% to two agents, 10.0% to three agents and fewer than 1% to four agents (supplementary figure). Exposure to the composite group VDGF was most prevalent (47.7%), followed by all solvents (29.7%), then metals (11.0%) and then all pesticides (3.5%) (figure 2).

**TABLE 1:** Summary of characteristics for the participants with spirometry and occupational data available in the UK Biobank

**Characteristics**	**Female**	**Male**	**Total**
**Participants n**	39 256	25 889	65 145
**Age at recruitment years**	56±8	57±8	56±8
**Ethnicity**			
White	36 723 (93.5)	24 450 (94.4)	61 173 (93.9)
Non-white	2533 (6.5)	1438 (5.6)	3971 (6.1)
**Smoking status**			
Never	23 980 (61.1)	13 529 (52.2)	37 509 (57.6)
Ex-smoker	13 021 (33.2)	10 280 (39.7)	23 301 (35.8)
Current smoker	2255 (5.7)	2080 (8.1)	4335 (6.6)
**Pack-years of smoking**	17.1±13.5	21.5±17.5	19.1±15.6
**Townsend deprivation index**	−2.5 (−3.8– −0.3)	−2.6 (−3.9– −0.6)	−2.5 (−3.9– −0.4)
**FEV_1_ L**	2.5 (2.2–2.8)	3.4 (3.0–3.9)	2.8 (2.4–3.3)
**FEV_3_ L**	3.0 (2.7–3.4)	4.1 (3.7–4.6)	3.4 (2.9–4.0)
**FEV_6_ L**	3.2 (2.9–3.6)	4.4 (4.0–5.0)	3.6 (3.1–4.3)
**FVC L**	3.3 (2.9–3.7)	4.5 (4.0–5.0)	3.6 (3.1–4.4)
**FEF_25–75_ L·s^−1^**	2.2 (1.7–2.7)	2.9 (2.2–3.5)	2.4 (1.9–3.0)
**FEV_1/_FEV_6_ <LLN**	5685 (14.5)	4307 (16.6)	9992 (15.3)
**FEV_3/_FEV_6_ <LLN**	8280 (21.1)	5662 (21.9)	13 942 (21.4)
**FEF_25–75_ <LLN**	2396 (6.1)	1253 (4.8)	3649 (5.6)

Data are presented as mean±sd, median (IQR) and n (%) unless otherwise indicated. FEV_1_: forced expiratory volume in 1 s; FEV_3_: forced expiratory volume in 3 s; FEV_6_, forced expiratory volume in 6 s; FVC: forced vital capacity; FEF_25–75_: mean forced expiratory flow between 25% and 75% of the forced vital capacity.

Univariable analyses showed significant associations between SAO (using both definitions) and lifetime occupational exposure to: VDGF and specifically to mineral dust, gases and fumes; all pesticides, and insecticides, herbicides and fungicides when considered as separate exposures; and metals. A significant association was observed for biological dust using FEF_25–75_ <LLN and FEV_3_/FEV_6_ <LLN. A significant association was also seen for lifetime exposure to aromatic solvents and FEV_3_/FEV_6_ <LLN but not for FEF_25–75_ <LLN (supplementary table).

In the multivariable analyses, significant associations remained for both parameters for highest exposures to all pesticides and insecticides and fungicides when considered individually. In most cases, there was an exposure–response observed with increasing cumulative pesticide exposure unit-years. There was also an inverse significant association between FEV_3_/FEV_6_ <LLN and highest exposure to aromatic solvents and metals, but no exposure–response association observed (table 2).

**TABLE 2:** Association of small airways obstruction with lifetime occupational exposures in UK Biobank

**ALOHA+ JEM agent**	**FEF_25–75_ <LLN**		**FEV_3_/FEV_6_ <LLN**	
	**OR (95% CI)**	**p-value**	**OR (95% CI)**	**p-value**
**VDGF**				
** **Low (<2 EU-years)	1.02 (0.93–1.11)	0.7	0.99 (0.95–1.05)	0.8
** **Moderate (2–17 EU-years)	1.06 (0.97–1.16)	0.2	0.98 (0.93–1.03)	0.4
** **High (≥18 EU-years)	0.85 (0.71–1.00)	0.06	0.98 (0.89–1.07)	0.6
**Biological dust**				
** **Low (<18 EU-years)	1.09 (0.99–1.19)	0.08	1.00 (0.98–1.03)	0.8
** **Moderate (18–44 EU-years)	1.07 (0.97–1.19)	0.2	1.04 (0.98–1.10)	0.2
** **High (≥45 EU-years)	0.95 (0.77–1.16)	0.6	0.94 (0.84–1.05)	0.3
**Mineral dust**				
** **Low (<2 EU-years)	1.05 (0.93–1.18)	0.05	0.95 (0.89–1.02)	0.1
** **Moderate (2–5 EU-years)	1.04 (0.91–1.19)	0.5	0.96 (0.89–1.03)	0.3
** **High (≥6 EU-years)	0.94 (0.73–1.19)	0.6	0.97 (0.85–1.11)	0.7
**Gases and fumes**				
** **Low (<2 EU-years)	1.00 (0.92–1.10)	1.0	0.98 (0.93–1.03)	0.5
** **Moderate (2–16 EU-years)	0.99 (0.90–1.09)	0.9	0.97 (0.92–1.03)	0.4
** **High (≥17 EU-years)	0.89 (0.74–1.06)	0.2	0.93 (0.84–1.02)	0.1
**All pesticides**				
** **Low (≤1 EU-years)	0.87 (0.66–1.12)	0.3	0.95 (0.82–1.09)	0.4
** **Moderate (2–15 EU-years)	0.99 (0.53–1.70)	1.0	1.08 (0.77–1.49)	0.7
** **High (≥16 EU-years)	1.38 (1.07–1.76)	0.01	1.23 (1.06–1.43)	0.007
**Insecticides**				
** **Low (<2 EU-years)	0.93 (0.70–1.21)	0.6	0.94 (0.80–1.10)	0.4
** **Moderate (2–15 EU-years)	1.07 (0.56–1.88)	0.8	1.25 (0.88–1.74)	0.2
** **High (≥16 EU-years)	1.40 (1.08–1.78)	0.008	1.22 (1.04–1.42)	0.01
**Herbicides**				
** **Low (<16 EU-years)	0.88 (0.34–1.85)	0.8	0.83 (0.52–1.28)	0.4
** **Moderate (16–31 EU-years)	1.60 (1.21–2.06)	0.001	1.32 (1.11–1.57)	0.001
** **High (≥32 EU-years)	0.72 (0.28–1.54)	0.4	1.01 (0.65–1.52)	1.0
**Fungicides**				
** **Low (≤1 EU-years)	0.91 (0.68–1.19)	0.5	0.90 (0.76–1.05)	0.2
** **Moderate (2–15 EU-years)	0.93 (0.41–1.84)	0.9	1.12 (0.73–1.64)	0.6
** **High (≥16 EU-years)	1.47 (1.12–1.88)	0.003	1.26 (1.07–1.47)	0.006
**Chlorinated solvents**				
** **Low (≤1 EU-years)	0.90 (0.78–1.04)	0.2	1.01 (0.94–1.09)	0.7
** **Moderate (2–16 EU-years)	1.03 (0.86–1.22)	0.7	0.99 (0.90–1.09)	0.9
** **High (≥17 EU-years)	0.96 (0.70–1.28)	0.8	0.85 (0.71–1.00)	0.06
**Aromatic solvents**				
** **Low (≤1 EU-years)	0.96 (0.85–1.09)	0.6	1.03 (0.96–1.10)	0.5
** **Moderate (2–3 EU-years)	0.84 (0.67–1.05)	0.1	1.07 (0.95–1.19)	0.3
** **High (≥4 EU-years)	0.92 (0.72–1.15)	0.5	0.85 (0.75–0.97)	0.02
**Other solvents**				
** **Low (≤1 EU-years)	1.00 (0.95–1.05)	0.6	0.97 (0.88–1.07)	1.0
** **Moderate (2–3 EU-years)	1.04 (0.97–1.11)	0.7	1.03 (0.91–1.16)	0.3
** **High (≥4 EU-years)	0.98 (0.90–1.08)	0.9	0.99 (0.83–1.16)	0.7
**Metals**				
** **Low (<2 EU-years)	0.93 (0.79–1.07)	0.3	1.02 (0.94–1.10)	0.6
** **Moderate (2–19 EU-years)	1.00 (0.81–1.22)	1.0	0.97 (0.87–1.08)	0.6
** **High (≥20 EU-years)	0.95 (0.67–1.32)	0.8	0.75 (0.62–0.91)	0.003

Model adjusted for age, sex, smoking status, smoking pack-years, UK Biobank assessment centre, Townsend deprivation index and ethnicity. p-values calculated by Wald test. FEF_25–75_: mean forced expiratory flow between 25% and 75% of the forced vital capacity; FEV_3_: forced expiratory volume in 3 s; FEV_6_, forced expiratory volume in 6 s; EU-years: exposure unit-years; VDGF: vapours, gases, dusts and fumes; LLN: lower limit of normal; JEM: job exposure matrix.

When accounting for co-exposures, SAO defined as FEV_3_/FEV_6_ <LLN was significantly associated with the highest cumulative exposure to all pesticides (OR 1.24, 95% CI 1.06–1.44) and negatively associated with the highest cumulative exposure to metals (OR 0.77, 95% CI 0.62–0.94) and the highest cumulative exposure to aromatic solvents (OR 0.85, 95% CI 0.73–0.99) (table 3).

**TABLE 3:** Association of small airways obstruction, defined as FEV_3_/FEV_6_ <LLN, with lifetime occupational exposures (multivariable model, adjusted for co-exposures)

**ALOHA+ JEM agent**	**FEV_3_/FEV_6_ <LLN**	
	**OR (95% CI)**	**p-value**
**All pesticides**		
** **Low (≤1 EU-years)	0.95 (0.82–1.10)	0.5
** **Moderate (2–15 EU-years)	1.06 (0.76–1.47)	0.7
** **High (≥16 EU-years)	1.24 (1.06–1.44)	0.008
**Aromatic solvents**		
** **Low (≤1 EU-years)	1.01 (0.93–1.10)	0.8
** **Moderate (2–3 EU-years)	1.03 (0.90–1.18)	0.7
** **High (≥4 EU-years)	0.85 (0.73–0.99)	0.04
**Other solvents**		
** **Low (≤1 EU-years)	0.99 (0.93–1.06)	0.8
** **Moderate (2–3 EU-years)	1.06 (0.98–1.15)	0.1
** **High (≥4 EU-years)	1.06 (0.95–1.17)	0.3
**Metals**		
** **Low (<2 EU-years)	1.01 (0.93–1.10)	0.8
** **Moderate (2–19 EU-years)	0.98 (0.87–1.10)	0.7
** **High (≥20 EU-years)	0.77 (0.62–0.94)	0.01

Model adjusted for age, sex, smoking status, smoking pack-years, UK Biobank assessment centre, Townsend deprivation index, ethnicity and the other exposures. p-values calculated by Wald test. FEV_3_: forced expiratory volume in 3 s; FEV_6_: forced expiratory volume in 6 s; LLN: lower limit of normal; EU-years: exposure unit-years; JEM: job exposure matrix.

## Discussion

In this large UK population-based study, the prevalence of SAO varied from 5.6% to 21.4% depending on the definition used, and SAO was consistently negatively associated with lifetime exposure to pesticides, particularly insecticides and fungicides, in an exposure–response manner.

Workers exposed to pesticides commonly report respiratory symptoms, but do not necessarily show abnormal lung function [26, 27]. Previous studies in the UK Biobank Cohort [28] and in other cohort studies [29, 30] have found associations between occupational pesticide exposure and airflow obstruction and COPD. However, a recent systematic review and meta-analysis of respiratory function and exposure to pesticides found only tentative evidence that exposure to cholinesterase-inhibiting pesticides reduced FEV_1_/FVC and no evidence that paraquat exposure affected lung function in farmers [31]. One possible mechanism explaining this relationship may involve differential DNA methylation [32]. We adjusted our models for several known and potential confounders. However, it is possible that the observed association between pesticide exposure and SAO is due to unmeasured confounding factors. Many of the workers exposed to pesticides will be farmers and agricultural workers who may also be exposed to agents that are established causes of airways diseases and which can cause SAO on spirometry. For example, crop farmers are likely exposed to grain dust, which is considered a cause of occupational asthma, while animal stock farmers may also be exposed to endotoxins, which has been associated with non-atopic asthma, and *Thermophilic actinomycetes* on mouldy hay, which has been linked to hypersensitivity pneumonitis [33, 34]. Our finding of an inverse relationship between SAO and the highest cumulative exposure to aromatic solvents and to metals was intriguing and is in contrast with other literature reporting both increased fixed airflow obstruction [35] and increased decline in FEV_1_ [36] with these exposures. We hypothesise that this is likely due to the healthy worker survivor bias, which may be more pronounced in those with heavy industrial exposures or due to those with heavy exposures being less likely to be included in the UK Biobank population.

The UK Biobank study included just over 120 000 individuals with a lifetime occupational history, but this included those with lower quality spirometry records and because of the intra-individual variability in small airways measurements we limited our analysis to those with high-quality spirometry. We were able to obtain high-quality spirometry from a large number of participants. The ALOHA+ JEM permits a semi-quantitative exposure assessment to be made and allowed stratification of cumulative exposure based on intensity and duration over a lifetime. The UK Biobank is a population cohort and minimises bias due to the healthy worker effect. Small airways measurements (FEF_25–75_ and FEV_3_) are dependent on performance of high-quality and reproducible spirometry. To reduce the chance of false positive cases of SAO we included only those with highest quality spirometry. Given our concerns about underestimation of FVC we defined SAO using FEF_25–75_ and FEV_3_/FEV_6_ but suggest that overall the best estimate of SAO in this population is FEV_3_/FEV_6_.

Despite these limitations, spirometry-derived parameters remain useful in large population-based studies for assessing SAO where other techniques would not be practical and have not been performed. Studies have shown good associations between these spirometric parameters and alternative methods of assessing SAO. Yee *et al.* [6] showed that FEV_3_/FEV_6_ <LLN was associated with lower FEV_1_, poorer health status, more emphysema and more functional small airways disease on quantitative imaging, and was also associated with development of COPD according to spirometry results (post-bronchodilator FEV_1_/FVC <0.7) during study follow-up. In another study by Qin *et al.* [37], individuals with SAO (defined using FEF_25–75_) in comparison to those with normal spirometry had more emphysema, a smaller airway lumen and larger airways walls in proportion to airway area. In addition, at the 9th generation of airway branching, FEF_25–75_ correlated with computed tomography (CT) scan markers of airways disease [37]. This is important as the 8–9th generation of airway branching is where the largest density of small airways is found. Finally, Lu *et al.* [38] compared IOS and spirometry to identify SAO and whilst there was only slight agreement (kappa 0.322, p<0.001), there was no significant difference in CT scan abnormalities between IOS-defined SAO and spirometry-defined SAO suggesting that spirometry is not inferior to other methods as a diagnostic tool.

One limitation of our study is that UK Biobank is not representative of the whole of the UK population on several sociodemographic (*e.g.* ethnicity) and health-related characteristics (*e.g.* smoking), with evidence of a “healthy volunteer” selection bias. Although UK Biobank is not appropriate for estimating population disease prevalence, its large size and array of exposure measures do provide an unparalleled resource for inference of associations between various exposures and diseases [39].

The identification of particular jobs and occupational exposures that increase the risk of SAO offers the opportunity to identify occupations that may contribute to subclinical lung damage during the working life, but which may be the precursor of more serious disease later in older age. Knowing the relationship of such exposures with SAO, and detection of SAO in the context of normal FEV_1_ and FVC, allows identification of an “at risk” group. This knowledge can be used to inform targeted preventative policies and monitoring strategies aiming at early intervention to reduce respiratory morbidity and mortality in working populations. Identification of SAO may offer a more effective tool for screening people who have early signs of disease in relation to workplace exposures. Spirometry is already used as part of routine respiratory health surveillance to identify early changes suggestive of asthma and other obstructive lung diseases and is widely available and relatively cheap to perform. Given the lack of consensus between different definitions of SAO [18] further work is required to establish the most clinically relevant spirometric parameter to identify SAO in the general population.

Further longitudinal studies of workers exposed to pesticides are required in order to infer causality and to understand mechanism better. Meanwhile, occupational exposure to pesticides should be kept as low as is practicable through substitution, engineering controls and use of personal protective equipment. We also propose that measurement of small airways could form part of the screening process in health surveillance.

### Conclusions

Occupational pesticide exposure is associated with SAO in the UK Biobank population cohort, highlighting the need for intervention strategies for primary prevention and to reduce progression to more severe lung disease.

## Supplementary material

10.1183/23120541.00650-2022.Supp1 **Please note:** supplementary material is not edited by the Editorial Office, and is uploaded as it has been supplied by the author.
Supplementary material 00650-2022.SUPPLEMENT
