# Supplementary material for: Occupational exposures and small airway obstruction in the UK Biobank Cohort
Source: ERJ Open Res. 2023 May 22;9(3):00650-2022. doi: 10.1183/23120541.00650-2022 (PMC10204826; doi:10.1183/23120541.00650-2022)
Supplement: Supplementary file 1 [file 00650-2022.SUPPLEMENT.pdf]

Supplementary Figure

Proportion of participants per number of exposures to groups of occupational agents (N=65,145) (JEM groups:VGDF (vapours, gases, dusts and fumes); all pesticides (combination of fungicides, herbicides and insecticides); all solvents (combination of aromatic, chlorinated and other solvents); metals)

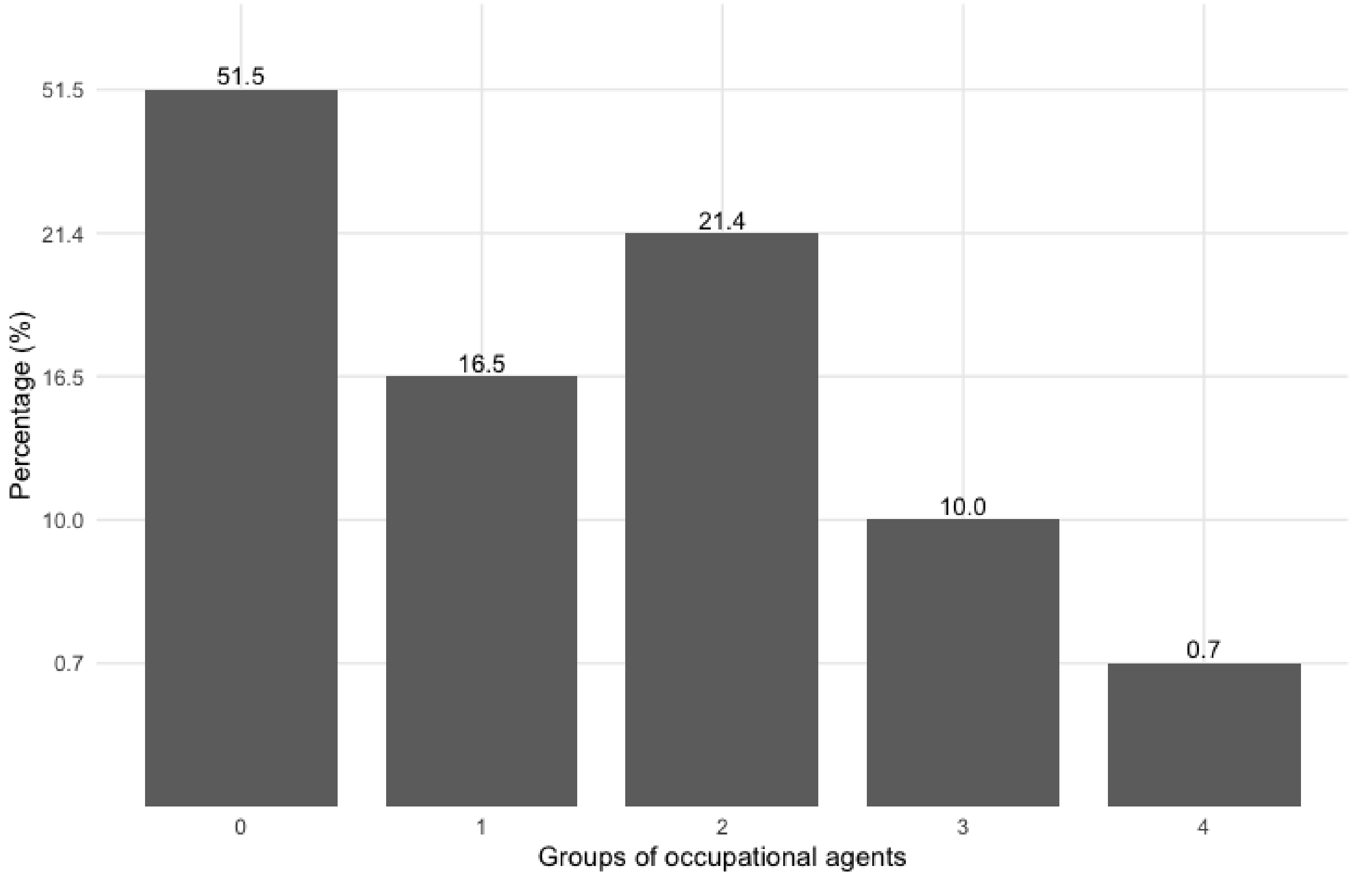

Supplementary Table

Univariable associations of Small Airway Obstruction with exposure to ALOHA+ JEM agents in participants with best quality spirometry and lifetime occupational history.

	FEF_{2575}<LLN		FEV_3/FEV_6<LLN	
**ALOHA+ JEM agent**	**OR (95% CI)**	***P***	**OR (95% CI)**	***P***
**VDGF**	1.13 (1.05-1.20)	**0.001**	1.04 (1.01-1.08)	**0.02**
**Biological dust**	1.21 (1.13-1.30)	**<0.001**	1.05 (1.01-1.09)	**0.03**
**Mineral dust**	1.14 (1.05-1.24)	**0.003**	1.09 (1.03-1.14)	**0.001**
**Gas and fumes**	1.09 (1.02-1.17)	**0.01**	1.06 (1.02-1.10)	**0.004**
**All pesticides**	1.22 (1.03-1.44)	**0.02**	1.16 (1.05-1.28)	**0.003**
**Insecticides**	1.28 (1.08-1.52)	**0.004**	1.16 (1.04-1.28)	**0.005**
**Herbicides**	1.46 (1.14-1.83)	**0.002**	1.25 (1.08-1.44)	**0.003**
**Fungicides**	1.29 (1.08-1.54)	**0.005**	1.16 (1.04-1.29)	**0.008**
**Chlorinated solvents**	0.93 (0.84-1.03)	0.1	1.04 (0.98-1.10)	0.2
**Aromatic solvents**	0.91 (0.82-1.00)	0.05	1.06 (1.00-1.12)	**0.03**
**Other solvents**	1.02 (0.95-1.10)	0.6	1.03 (0.99-1.07)	0.2
**Metals**	0.88 (0.79-0.99)	**0.03**	1.06 (1.00-1.13)	**0.04**

OR: odds ratio; 95% CI: 95% confidence intervals; FEV3: forced expiratory volume in three seconds; FEV_6, forced expiratory volume in six seconds; FEF25-75: mean forced expiratory flow between 25 and 75% of the forced vital capacity; VGDF: vapours, gases, dusts and fumes; LLN: lower limit of normal; P-value, Wald test.
